# Cardiovascular risk factors in gout, psoriatic arthritis, rheumatoid arthritis and ankylosing spondylitis: a cross-sectional survey of patients in Western Sweden

**DOI:** 10.1136/rmdopen-2021-001568

**Published:** 2021-05-23

**Authors:** Anton Jonatan Landgren, Mats Dehlin, Lennart Jacobsson, Ulrika Bergsten, Eva Klingberg

**Affiliations:** 1 Department of Rheumatology and Inflammation Research, Institute of Medicine, Sahlgrenska Academy, University of Gothenburg, Sweden; 2 Region Västra Götaland, Research and Development Primary Health Care, Gothenburg, Sweden; 3 R&D Department at Region Halland, Halmstad, Sweden

**Keywords:** ankylosing spondylitis, epidemiology, gout, psoriatic arthritis, rheumatoid arthritis

## Abstract

**Objectives:**

We aimed to compare traditional (trad) cardiovascular risk factors (CVRFs) among patients with gout, psoriatic arthritis (PsA), rheumatoid arthritis (RA) and ankylosing spondylitis (AS) stratified by sex.

**Methods:**

A survey was sent to patients with gout (n=1589), PsA (n=1200), RA (n=1246) and AS (n=1095). Patients were retrieved from Sahlgrenska University Hospital, the hospitals of Uddevalla and Skövde, and 12 primary care centres in Western Sweden. The prevalence of self-reported trad-CVRFs was compared between diagnoses by age standardisation with the 2018 population of Sweden as the standard population.

**Results:**

In total, 2896 (56.5%) of 5130 patients responded. Hypertension was the most frequently found comorbidity, reported by 65% of patients with gout, 41% with PsA, 43% with RA and 29% with AS. After age standardisation, women and men with gout had significantly more obesity (body mass index ≥30 kg/m^2^), hypertension, diabetes, hyperlipidaemia and multiple trad-CVRFs, compared with those with PsA, RA and AS. Obesity was significantly more common in PsA than in RA. In women, obesity, hypertension and multiple trad-CVRFs were more frequently reported in PsA than in RA and AS, whereas similar prevalence of CVRFs and coexistence of multiple trad-CVRFs were found in men with PsA, RA and AS.

**Conclusions:**

Women and men with gout had the highest prevalence of trad-CVRFs. Differences in occurrence of CVRFs by sex were found in patients with PsA, RA and AS. In women, patients with PsA had higher occurrence of trad-CVRFs than those with RA and AS, whereas in men the distribution of CVRFs was similar in PsA, RA and AS.

**Key messages**

**What is already known about this subject?**

- Cardiovascular risk factors (CVRFs) are over-represented in patients with inflammatory joint diseases (IJDs) such as gout, psoriatic arthritis (PsA), rheumatoid arthritis (RA) and ankylosing spondylitis (AS), but no study has compared the prevalence of CVRF in these diseases in the same setting.

**What does this study add?**

- In this study, we found high prevalence of CVRFs across IJDs. In particular, patients with gout and PsA (especially women) reported high prevalence of CVRFs.
- More than 70% of patients with IJDs reported at least one CVRF, and multiple CVRFs were frequently present in patients with IJDs, especially in patients with gout of both sexes and in women with PsA.

**How might this impact on clinical practice or future developments?**

- This study raises the awareness of screening for and treating CVRFs in patients with IJDs that already are at an increased risk of cardiovascular events.

## Background

Gout, psoriatic arthritis (PsA), rheumatoid arthritis (RA) and ankylosing spondylitis (AS) are common rheumatic diseases characterised by acute and chronic inflammation of peripheral joints, entheses or spine. All inflammatory joint diseases (IJDs) are associated with joint stiffness and pain, increasing the risk of physical inactivity, depression, overeating and metabolic syndrome (MetS), comprising obesity, hypertension, hyperlipidaemia and diabetes. It is well known that patients with IJDs have an increased risk of cardiovascular disease (CVD).^1^ For PsA and AS, HRs for acute coronary syndrome have been reported at 1.76 and 1.54, respectively, compared with the general population in a Swedish nationwide cohort study.^2^ The increased risk of CVD in patients with IJDs has been suggested to depend on a combination of accelerated atherosclerotic disease, due to inflammation and increased occurrence of traditional (trad) cardiovascular risk factors (CVRFs). Previous studies have separately reported on an increased prevalence of trad-CVRFs, compared with the normal population, in gout,^3 4^ PsA^5–10^ RA^8 9^ and AS.^9 11^ Differences in the occurrence of trad-CVRFs between sexes have been noted in the general population^12^ and for gout,^3^ PsA^10^ and RA.^13^ In AS, higher risk of death from CVD has been noted in men compared with women.^14^ In a study investigating a large number of modifiable risk factors in the general population, more than 90% of the risk for acute myocardial infarction was accounted for by trad-CVRFs.^15^ In a meta-analysis by Baghdadi *et al*, trad-CVRFs seemed to have as great impact on CV morbidity in RA as in the general population.^16^ Risk factors for CVD, such as smoking and obesity, may also increase the risk of some IJDs by being part of the pathogenesis. For example, smoking is a well-established risk factor for anti-citrullinated-protein antibody (ACPA)-positive RA.^17^ Obesity is a well-known risk factor for gout,^18^ psoriasis^19^ and PsA.^20^ If obesity and other components associated with MetS are more common in some IJDs, it can provide support for a pathophysiological connection between obesity/MetS and these diseases. The burden of CVRFs varies between different IJDs, and this calls for comparative studies assessing the risk across diseases.^21^ Occurrence of trad-CVRFs varies between IJDs, which may reflect unwanted differences in identification and treatment of trad-CVRFs between IJDs. A comparison taking into account the large variations in age and sex between different IJDs is therefore warranted. To the best of our knowledge, no study has included the most common IJD (gout) in the comparison between IJDs and no study has compared CVRFs among the common IJDs gout, PsA, RA and AS in the same setting. We aimed to assess and compare the occurrence of trad-CVRFs^16^ (obesity, smoking, physical inactivity, hypertension, diabetes, hyperlipidaemia), as well as coexistence of multiple trad-CVRFs in patients with gout, PsA, RA and AS, stratified by sex.

## Methods

### Setting

We performed a cross-sectional questionnaire study in Western Sweden.

### Study population

**Figure 1:**
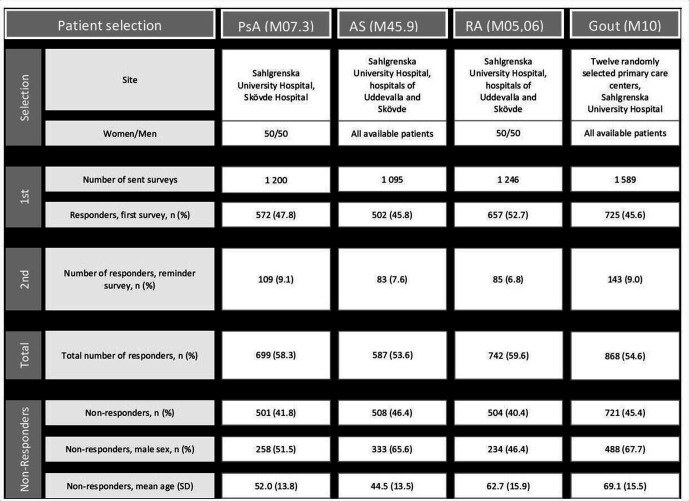
Patient selection. AS, ankylosing spondylitis; PsA, psoriatic arthritis; RA, rheumatoid arthritis.

All individuals who were aged ≥18 years and had at least one International Classification of Diseases, 10th Revision (ICD-10) diagnosis of gout (ICD-10 codes M10), PsA (ICD-10 codes M073), RA (ICD 10 codes M05 or M06) or AS (ICD 10 codes M459), recorded at a healthcare visit to a physician at a rheumatology clinic (for all patients) or a primary care centre (exclusively for gout patients) during a 2-year period (January 2015 through February 2017), were identified. The first questionnaire and the reminder questionnaire were sent during the year 2017. Patients were recruited from the Department of Rheumatology at Sahlgrenska University Hospital, the hospitals of Uddevalla and Skövde, and for gout from 12 primary care centres in Western Sweden and Sahlgrenska University Hospital. The vast majority (91%) of patients with gout were recruited from primary care centres. From these groups of patients, randomly selected patients with PsA (n=1200) and RA (n=1246), with a predefined sex distribution of 50% women and 50% men, were sent a postal questionnaire. This distribution was chosen to enable analyses among diagnoses by age and sex. All identified patients with gout (n=1589) and AS (n=1095) were sent a questionnaire. A reminder survey was sent to those who did not reply to the initial questionnaire. Recruitment of patients and non-responder data are further described in figure 1. The questionnaire included questions on demographics, trad-CVRFs (obesity, smoking, physical inactivity, hypertension, diabetes, hyperlipidaemia) and current medication. Returning the questionnaire was considered informed consent, and all participants were informed in writing that the reported data would be published. The Regional Ethical Review Board in Gothenburg, Sweden, approved the study (23 August 2016, Dnr 519-16). The study was carried out in accordance with the Declaration of Helsinki.

### Definition of variables

Body mass index (BMI, kg/m^2^) was calculated using self-reported weight and height. Obesity was defined as BMI ≥30.0 kg/m^2^ in accordance with the WHO standard. Physical activity was divided into ≤3 hours per week or >3 hours per week (which is above the WHO and the Swedish recommendation of at least 150 min per week of moderate-intensity physical activity for adults aged 18–64 years^22^). A person was considered living a sedentary lifestyle if the reported daily total sitting time during a normal day, excluding time spent sleeping, was ≥7 hours^23^ in combination with ≤3 hours of physical activity per week.

Smoking status was characterised as ‘current’, ‘ever’ or ‘never’ smoker. Furthermore, the patients were asked whether they had hypertension, hyperlipidaemia or diabetes. Obesity, smoking, having a sedentary lifestyle, hypertension, diabetes and hyperlipidaemia were defined as trad-CVRFs.

Highest completed education was assessed and divided into ‘≤12 years’ or ‘>12 years’.

To assess multiple trad-CVRFs, composite variables containing self-reported obesity, hypertension, diabetes, hyperlipidaemia, current smoking and having a sedentary lifestyle were created. The patients were grouped according to a minimum of one to four trad-CVRFs.

Disease-modifying antirheumatic drugs (DMARDs) were grouped into either conventional synthetic (cs) or biologic (b) DMARDs.

## Statistics

**Figure 2:**
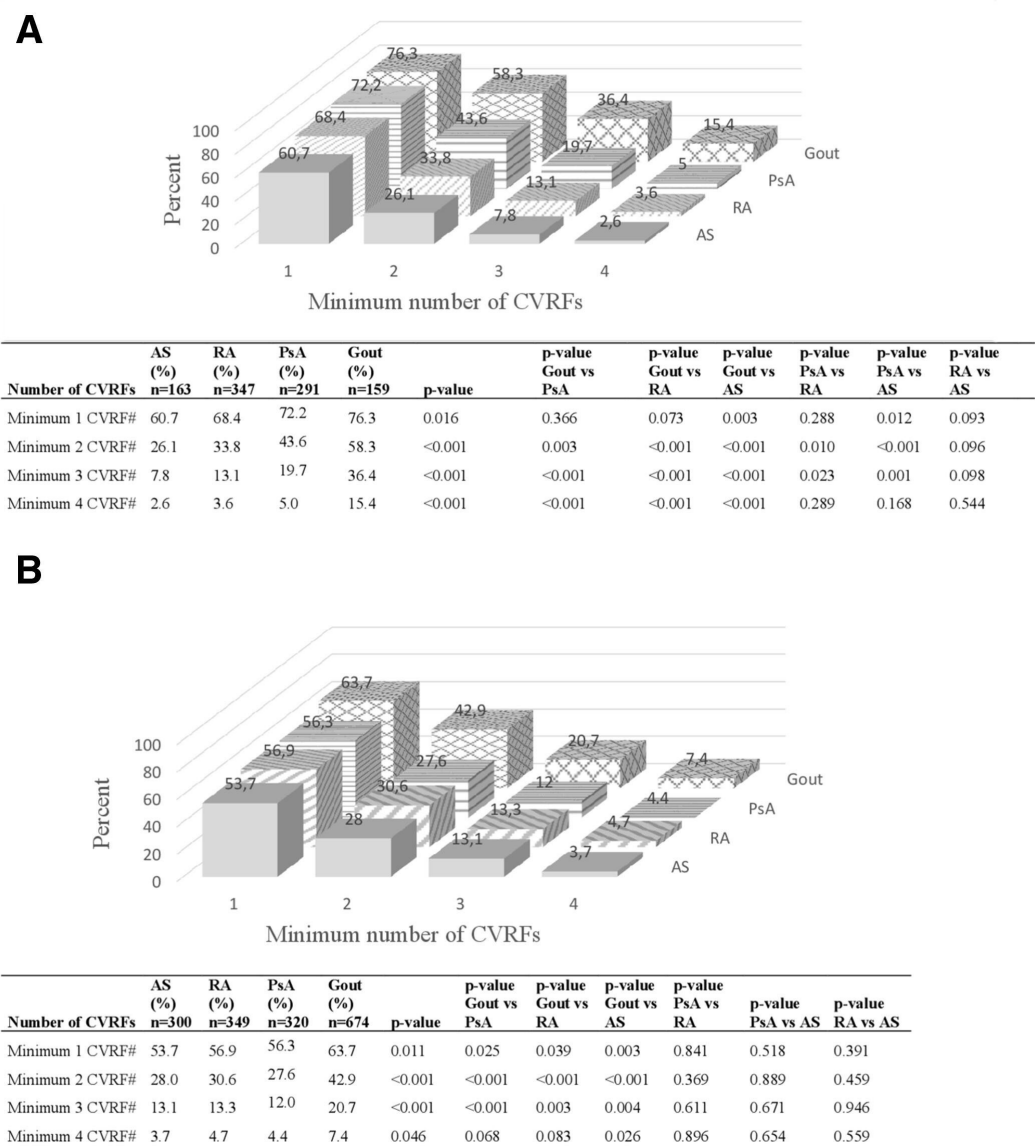
(A) Age-standardised prevalence and comparison between different IJDs for different levels of multiple traditional (trad) CVRFs in women (ages 45–89 years, n=960). (B) Age-standardised prevalence and comparison between different IJDs for different levels of multiple trad-CVRFs in men (ages 30–89 years, n=1643). AS, ankylosing spondylitis; CVRF, cardiovascular risk factor; IJD, inflammatory joint disease; PsA, psoriatic arthritis; RA, rheumatoid arthritis. # Body mass index ≥30 kg/m^2^, hypertension, diabetes, hyperlipidaemia, current smoking, total sitting time ≥7 hours per day+physical activity ≤3 hours per week.

Data were expressed as absolute counts and proportions for categorical variables and as means±SD for continuous variables. χ^2^ tests were used for comparisons of categorical variables and to compare standardised rates/prevalence between different IJD diagnoses. Independent-samples t-test was used for comparisons of mean values for continuous variables. For comparisons of mean values across more than two groups, analysis of variance (ANOVA) was used. Due to differences in age between patient groups with different IJDs, age standardisation was performed for selected variables (figure 2A, B). For women, age-standardised calculations were restricted to ages 45–89 years, because only few women with gout below the age of 45 years were included in the study. The Swedish population of 2018 was retrieved from Statistics Sweden, divided into 5-year age intervals and was used as the standard population. All tests were two-tailed, and p values <0.05 were considered significant. SPSS Statistics V.25 (IBM) was used for statistical analyses.

## Results

Characteristics of the study populations, stratified by diagnosis and sex, are presented in tables 1–3. A total of 868 patients with gout (79.6% men), 699 patients with PsA (46.9% men), 742 patients with RA (47.8% men) and 587 patients with AS (56.4% men) responded to the questionnaire. The response rates were as follows: 54.6% for patients with gout, 58.3% for those with PsA, 59.6% for those with RA and 53.6% for those with AS. Patients with RA had significantly higher response rate compared with those with gout (p=0.0086) and AS (p=0.004). Non-responders were significantly younger than responders for all diagnoses (age mean±SD, 58.2±17.7 vs 62.5±15.3, p<0.01). Non-responders with AS were more frequently men (65.6%) compared with responders (56.4%), p=0.002, whereas non-responders with gout were more frequently women (32.3%) compared with responders (20.4%), p<0.001 (non-responder data are shown in figure 1 and online supplemental table 1). Patients with gout were significantly older than patients with PsA, RA and AS (table 1). More than half of the patients with PsA and RA reported use of cs-DMARD. Use of b-DMARDs was reported by 22%–36% of patients with PsA, RA and AS. In PsA and AS, tumour necrosis factor inhibitors comprised the vast majority of b-DMARDS. Urate-lowering therapy was reported by 49% of patients with gout. The medications of the study population are given in tables 1–3 and reflect differences in comorbidities between the IJDs.

10.1136/rmdopen-2021-001568.supp1 Supplementary data

**Table 1:** Characteristics and prevalence of traditional CVRFs in the entire survey population (n=2896)

	Gout	PsA	RA	AS	P value
Total (n)	868	699	742	587	
Male sex, n (%)	691 (79.6)	328 (46.9)	355 (47.8)	331 (56.4)	<0.001
Age, mean (SD)	71.3 (11.9)	56.6 (13.2)	66.8 (13.1)	51.1 (14.8)	<0.001
Age groups, n (%)					<0.001
20–39	13 (1.5)	75 (10.7)	35 (4.7)	139 (23.8)	
40–59	118 (13.6)	327 (46.8)	151 (20.4)	254 (43.5)	
60–79	521 (60.0)	277 (39.6)	457 (61.6)	182 (31.2)	
80+	216 (24.9)	20 (2.9)	99 (13.3)	9 (1.5)	
Education, n (%)					
≤12 years	526 (60.6)	388 (55.5)	492 (66.3)	281 (47.9)	<0.001
>12 years	311 (35.8)	298 (42.6)	237 (31.9)	293 (49.9.)	<0.001
BMI, mean (SD)	28.2 (4.6)	27.4 (4.6)	26.2 (4.3)	26.2 (4.6)	<0.001
Obesity, BMI ≥30.0 kg/m^2^, n (%)	214 (24.7)	161 (23.0)	113 (15.2)	100 (17.0)	<0.001
Smoking, n (%)					
Never	392 (45.2)	320 (45.8)	312 (42.0)	310 (52.8)	0.001
Ever	443 (51.0)	370 (52.9)	420 (56.6)	268 (45.7)	0.001
Current	49 (5.6)	83 (11.9)	93 (12.5)	61 (10.4)	<0.001
Physical activity, n (%)					
≤3 hours per week	499 (57.5)	337 (48.2)	408 (55.0)	260 (44.3)	<0.001
>3 hours per week	334 (38.5)	351 (50.2)	322 (43.4)	319 (54.3)	<0.001
Total sitting time, n (%)					
<7 hours per day	469 (54.0)	418 (59.8)	473 (63.7)	342 (58.3)	0.001
≥7 hours per day	399 (46.0)	281 (40.2)	269 (36.3)	245 (41.7)	0.001
Sedentary§, n (%)	276 (31.8)	158 (22.6)	184 (24.8)	131 (22.3)	<0.001
Hypertension, n (%)	561 (64.6)	283 (40.5)	322 (43.4)	172 (29.3)	<0.001
Diabetes, n (%)	197 (22.7)	72 (10.3)	80 (10.8)	28 (4.8)	<0.001
Hyperlipidaemia, n (%)	279 (32.1)	121 (17.3)	140 (18.9)	64 (10.9)	<0.001
Minimum number of CVRFs*, n (%)					
Minimum 1 CVRF*	719 (82.8)	481 (68.8)	524 (70.6)	339 (57.8)	<0.001
Minimum 2 CVRFs*	498 (57.4)	248 (35.5)	269 (36.3)	145 (24.7)	<0.001
Minimum 3 CVRFs*	254 (29.3)	110 (15.7)	101 (13.6)	52 (8.9)	<0.001
Minimum 4 CVRFs*	88 (10.1)	34 (4.9)	31 (4.2)	16 (2.7)	<0.001
cs-DMARD†, n (%)	N/A	450 (64.4)	554 (74.7)	147 (25.0)	<0.001
b-DMARD‡, n (%)	2 (0.2)	173 (24.7)	166 (22.4)	210 (35.8)	<0.001
NSAIDs, n (%)	61 (7.0)	275 (39.3)	215 (29.0)	291 (49.6)	<0.001
Glucocorticoids, n (%)	30 (3.5)	54 (7.7)	167 (22.5)	27 (4.6)	<0.001
Antihypertensives, n (%)	575 (66.2)	239 (34.2)	330 (44.5)	144 (24.5)	<0.001
Antidiabetics, n (%)	164 (18.9)	51 (7.3)	62 (8.4)	21 (3.6)	<0.001
Lipid lowering, n (%)	290 (33.4)	101 (14.4)	155 (20.9)	53 (9.0)	<0.001
Urate-lowering therapy (allopurinol), n (%)	428 (49.3)	N/A	N/A	N/A	

Comparisons across diagnoses with analysis of variance and χ^2^ tests.

*BMI ≥30 kg/m^2^, hypertension, diabetes, hyperlipidaemia, current smoking, total sitting time ≥7 hours per day+physical activity ≤3 hours per week.

†Salazopyrin, methotrexate, Arava and apremilast.

‡Tumour necrosis factor inhibitors, tocilizumab, sekukinumab, rituximab, abatacept, anakinra and ustekinumab.

§Total sitting time ≥7 hours per day+physical activity ≤3 hours per week.

AS, ankylosing spondylitis; b-DMARD, biologic DMARD; BMI, body mass index; cs-DMARD, conventional synthetic DMARD; CVRF, cardiovascular risk factor; DMARD, disease-modifying antirheumatic drug; NSAIDs, non-steroidal anti-inflammatory drugs ATC-code (M01A); PsA, psoriatic arthritis; RA, rheumatoid arthritis.;

**Table 2:** Characteristics and prevalence of traditional CVRFs in women in the survey population (n=1191)

	Gout	PsA	RA	AS	P value
Total (n)	177	371	387	256	
Age, mean (SD)	75.3 (11.7)	56.7 (14.0)	65.6 (14.1)	50.5 (14.4)	<0.001
Age groups, n (%)					<0.001
20–39	2 (1.1)	43 (11.6)	22 (5.7)	62 (23.4)	
40–59	15 (8.5)	161 (43.4)	96 (24.8)	117 (45.7)	
60–79	94 (53.1)	154 (41.5)	215 (55.6)	73 (28.5)	
80+	66 (37.3)	13 (3.5)	54 (14.0)	2 (0.8)	
Education, n (%)					
≤12 years	119 (67.2)	184 (49.6)	233 (60.2)	103 (40.2)	<0.001
>12 years	44 (24.9)	181 (48.8)	146 (37.7)	147 (57.4)	<0.001
BMI, mean (SD)	29.0 (5.3)	27.5 (5.2)	25.9 (4.6)	25.8 (5.1)	<0.001
Obesity, BMI ≥30.0 kg/m^2^, n (%)	60 (33.9)	103 (27.8)	64 (16.5)	44 (17.2)	<0.001
Smoking, n (%)					
Never	97 (54.8)	147 (39.6)	182 (47.0)	152 (59.4)	<0.001
Ever	67 (37.9)	218 (58.8)	198 (51.2)	100 (39.1)	<0.001
Current	9 (5.1)	58 (15.6)	51 (13.2)	19 (7.4)	0.001
Physical activity, n (%)					
≤3 hours per week	106 (59.9)	188 (50.7)	224 (57.9)	119 (46.5)	<0.001
>3 hours per week	54 (30.5)	176 (47.4)	155 (40.1)	134 (52.3)	<0.001
Total sitting time, n (%)					
<7 hours per day	99 (55.9)	238 (64.2)	252 (65.1)	161 (62.9)	0.895
≥7 hours per day	63 (35.6)	126 (34.0)	129 (33.3)	92 (35.9)	0.895
Sedentary§, n (%)	49 (27.7)	77 (20.8)	95 (24.5)	54 (21.1)	0.233
Hypertension, n (%)	118 (66.7)	147 (39.6)	159 (41.1)	68 (26.6)	<0.001
Diabetes, n (%)	40 (22.6)	38 (10.2)	28 (7.2)	10 (3.9)	<0.001
Hyperlipidaemia, n (%)	61 (34.5)	65 (17.5)	65 (16.8)	23 (9.0)	<0.001
Minimum number of CVRF*, n (%)					
Minimum 1 CVRF*	144 (81.4)	258 (69.5)	267 (69.0)	142 (55.5)	<0.001
Minimum 2 CVRFs*	108 (61.0)	143 (38.5)	132 (34.1)	54 (21.1)	<0.001
Minimum 3 CVRFs*	61 (34.5)	65 (17.5)	47 (12.1)	16 (6.3)	<0.001
Minimum 4 CVRFs*	19 (10.7)	19 (5.1)	14 (3.6)	5 (2.0)	<0.001
cs-DMARD†, n (%)	N/A	221 (59.6)	281 (72.6)	62 (24.2)	<0.001
b-DMARD‡, n (%)	N/A	80 (21.6)	90 (23.3)	76 (29.7)	0.055
NSAIDs, n (%)	14 (7.9)	164 (44.2)	115 (29.7)	131 (51.2)	<0.001
Glucocorticoids, n (%)	7 (4.0)	38 (10.2)	85 (22.0)	18 (7.0)	<0.001
Antihypertensives, n (%)	119 (67.2)	128 (34.5)	151 (39.0)	56 (21.9)	<0.001
Antidiabetics, n (%)	33 (18.6)	27 (7.3)	20 (5.2)	10 (3.9)	<0.001
Lipid lowering, n (%)	54 (30.5)	50 (13.5)	60 (15.5)	20 (7.8)	<0.001
Urate-lowering therapy (allopurinol), n (%)	87 (49.2)	N/A	N/A	N/A	

Comparisons across diagnoses with analysis of variance and χ^2^ tests.

*BMI ≥30 kg/m^2^, hypertension, diabetes, hyperlipidaemia, current smoking, total sitting time ≥7 hours per day+physical activity ≤3 hours per week.

†Salazopyrin, methotrexate, Arava and apremilast.

‡Tumour necrosis factor inhibitors, tocilizumab, sekukinumab, rituximab, abatacept, anakinra and ustekinumab.

§Total sitting time ≥7 hours per day+physical activity ≤3 hours per week.

AS, ankylosing spondylitis; b-DMARD, biologic DMARD; BMI, body mass index; cs-DMARD, conventional synthetic DMARD; CVRF, cardiovascular risk factor; DMARD, disease-modifying antirheumatic drug; NSAIDs, non-steroidal anti-inflammatory drugs ATC-code (M01A); PsA, psoriatic arthritis; RA, rheumatoid arthritis.;

**Table 3:** Characteristics and prevalence of traditional CVRFs in men in the survey population (n=1705)

	Gout	PsA	RA	AS	P value
Total (n)	691	328	355	331	
Age, mean (SD)	70.3 (11.7)	56.3 (12.2)	68.1 (11.8)	51.6 (15.0)	<0.001
Age groups, n (%)					<0.001
20–39	11 (1.6)	32 (9.8)	13 (3.7)	77 (23.3)	
40–59	103 (14.9)	166 (50.6)	55 (15.5)	137 (41.5)	
60–79	427 (61.8)	123 (37.5)	242 (68.2)	109 (33.0)	
80+	150 (21.7)	7 (2.1)	45 (12.7)	7 (2.1)	
Education, n (%)					
≤12 years	407 (58.9)	204 (62.2)	259 (73.0)	178 (53.8)	<0.001
>12 years	267 (38.6)	117 (35.7)	91 (25.6)	146 (44.1)	<0.001
BMI, mean (SD)	28.0 (4.4)	27.2 (3.7)	26.6 (3.8)	26.5 (4.1)	<0.001
Obesity, BMI ≥30.0 kg/m^2^, n (%)	154 (22.3)	58 (17.7)	49 (13.8)	56 (16.9)	0.004
Smoking, n (%)					
Never	295 (42.7)	173 (52.7)	130 (36.6)	158 (47.7)	<0.001
Ever	376 (54.4)	152 (46.3)	222 (62.5)	168 (50.8)	<0.001
Current	40 (5.8)	25 (7.6)	42 (11.8)	42 (12.7)	<0.001
Physical activity, n (%)					
≤3 hours per week	393 (56.9)	149 (45.4)	184 (51.8)	141 (42.6)	<0.001
>3 hours per week	280 (40.5)	175 (53.4)	167 (47.6)	185 (55.9)	<0.001
Total sitting time, n (%)					
<7 hours per day	338 (48.9)	170 (51.8)	210 (59.2)	174 (52.6)	0.040
≥7 hours per day	336 (48.6)	155 (47.3)	140 (39.4)	153 (46.2)	0.040
Sedentary§, n (%)	227 (32.9)	81 (24.7)	89 (25.1)	77 (23.3)	0.002
Hypertension, n (%)	443 (64.1)	136 (41.5)	163 (45.9)	104 (31.4)	<0.001
Diabetes, n (%)	157 (22.7)	34 (10.4)	52 (14.6)	18 (5.4)	<0.001
Hyperlipidaemia, n (%)	218 (31.5)	56 (17.1)	75 (21.1)	41 (12.4)	<0.001
Minimum number of CVRF*, n (%)					
Minimum 1 CVRF*	575 (83.2)	223 (68.0)	257 (72.4)	197 (59.5)	<0.001
Minimum 2 CVRFs*	390 (56.4)	105 (32.0)	137 (38.6)	91 (27.5)	<0.001
Minimum 3 CVRFs*	193 (27.9)	45 (13.7)	54 (15.2)	36 (10.9)	<0.001
Minimum 4 CVRFs*	69 (10.0)	15 (4.6)	17 (4.8)	11 (3.3)	<0.001
cs-DMARD†, n (%)	N/A	229 (69.8)	273 (76.9)	85 (25.7)	<0.001
b-DMARD‡, n (%)	2 (0.3)	93 (28.4)	76 (21.4)	134 (40.5)	<0.001
NSAIDs, n (%)	47 (6.8)	111 (33.8)	100 (28.2)	160 (48.3)	<0.001
Glucocorticoids, n (%)	23 (3.3)	16 (4.9)	82 (23.1)	9 (2.7)	<0.001
Antihypertensives, n (%)	456 (66.0)	111 (33.8)	179 (50.4)	88 (26.6)	<0.001
Antidiabetics, n (%)	131 (19.0)	24 (7.3)	42 (11.8)	11 (3.3)	<0.001
Lipid lowering, n (%)	236 (34.2)	51 (15.5)	95 (26.8)	33 (10.0)	<0.001
Urate-lowering therapy (allopurinol), n (%)	341 (49.3)	N/A	N/A	N/A	

Comparisons across diagnoses with analysis of variance and χ^2^ tests

*BMI ≥30 kg/m^2^, hypertension, diabetes, hyperlipidaemia, current smoking, total sitting time ≥7 hours per day+physical activity ≤3 hours per week.

†Salazopyrin, methotrexate, Arava and apremilast.

‡Tumour necrosis factor inhibitors, tocilizumab, sekukinumab, rituximab, abatacept, anakinra and ustekinumab.

§Total sitting time ≥7 hours per day+physical activity ≤3 hours per week.

AS, ankylosing spondylitis; b-DMARD, biologic DMARD; BMI, body mass index; cs-DMARD, conventional synthetic DMARD; CVRF, cardiovascular risk factor; DMARD, disease-modifying antirheumatic drug; NSAIDs, non-steroidal anti-inflammatory drugs ATC-code (M01A); PsA, psoriatic arthritis; RA, rheumatoid arthritis.

### Prevalence of trad-CVRFs among patients with IJDs

Obesity was more prevalent in patients with gout (24.7%), closely followed by PsA (23.0%). In patients with AS and RA, 17.0% and 15.2%, respectively, reported obesity. Very few patients were underweight (BMI <18.5 kg/m^2^), in total 17 of 2896 patients. Hypertension was the most common trad-CVRF in all IJDs, reported by 64.6% of patients with gout, 43.4% with RA, 40.5% with PsA and 29.3% with AS. The second most prevalent trad-CVRF was hyperlipidaemia, reported by 32.1% of patients with gout, 18.9% with RA and 17.3% with PsA, but only by 10.9% with AS. Current smoking was reported by 12.5% of patients with RA, 11.9% with PsA, 10.4% with AS and 5.6% with gout. A sedentary lifestyle was reported by 31.8% of patients with gout, 24.8% with RA, 22.6% with PsA and 22.3% with AS. Occurrence of multiple trad-CVRFs was overall more common in gout, followed by PsA and RA and less common in patients with AS. Patients with gout more frequently presented with a minimum of one to four trad-CVRFs compared with other IJDs both before (tables 1–3) and after standardising for age (figure 2A, B).

### Age-standardised prevalence of trad-CVRFs by sex among patients with IJDs

After standardising for age, women with gout presented significantly higher prevalence of obesity, diabetes, hyperlipidaemia and a sedentary lifestyle compared with other IJDs, and hypertension was more prevalent compared with RA and AS. Women with gout also had the highest occurrence of multiple trad-CVRFs compared with other IJDs (figure 2A). Women with PsA did, however, more frequently report obesity and hypertension compared with women with RA and AS, and more diabetes and hyperlipidaemia than women with AS. Coherently, the level of ≥2 and ≥3 CVRFs was significantly higher in women with PsA compared with women with RA and AS, who had similar levels (figure 2A). The prevalence of a sedentary lifestyle was, however, similar in women with PsA, RA and AS. Women with AS had the lowest prevalence of current smoking, which otherwise was similar among all IJDs. Educational level was also similar in women in all IJDs (table 4A).

**Table 4:** Age-standardized prevalence of traditional CVRFs in women and men, comparisons across diagnoses are done with χ^2^ tests

	Gout (%) n=159	PsA (%) n=291	RA (%) n=347	AS (%) n=163	P value	P value gout vs PsA	P value gout vs RA	P value gout vs AS	P value PsA vs RA	P value PsA vs AS	P value RA vs AS
A: Age-standardised prevalence of traditional CVRFs in women, ages 45–89 years (n=960)											
Education ≤12 years	52.6	54.4	58.5	53.3	0.534	0.766	0.232	0.922	0.286	0.850	0.276
BMI ≥30 kg/m^2^	38.7	28.8	17.9	17.5	<0.001	0.028	<0.001	<0.001	<0.001	0.009	0.983
Current smoker	12.8	13.9	13.3	5.5	0.048	0.728	0.834	0.027	0.857	0.007	0.009
Ever smoker	40.0	59.2	49.9	50.0	0.001	<0.001	0.044	0.070	0.012	0.070	0.924
Physical activity ≤3 hours per week	60.9	50.4	59.1	47.3	0.012	0.033	0.681	0.013	0.030	0.503	0.012
Sedentary*	34.7	19.0	23.4	21.0	0.002	<0.001	0.008	0.006	0.172	0.614	0.531
Hypertension	52.9	46.9	40.6	36.0	0.009	0.216	<0.001	0.003	<0.001	0.030	0.338
Diabetes	18.1	11.5	7.4	5.4	<0.001	0.042	<0.001	<0.001	0.095	0.040	0.412
Hyperlipidaemia	30.6	21.2	16.6	12.4	<0.001	0.025	<0.001	<0.001	0.139	0.016	0.193

	Gout (%) n=674	PsA (%) n=320	RA (%) n=349	AS (%) n=300	P value	P value Gout vs PsA	P value Gout vs RA	P value Gout vs AS	P value PsA vs RA	P value PsA vs AS	P value RA vs AS
B: Age-standardised prevalence of traditional CVRFs in men, ages 30–89 years (n=1643)											
Education ≤12 years	50.7	56.1	72.0	53.5	<0.001	0.113	<0.001	0.450	<0.001	0.493	<0.001
BMI ≥30	18.9	11.8	10.6	14.2	0.001	0.006	<0.001	0.087	0.602	0.364	0.149
Current smoker	10.7	7.1	15.3	12.5	0.009	0.074	0.035	0.375	<0.001	0.021	0.343
Ever smoker	53.0	53.5	62.1	57.6	0.035	0.963	0.006	0.174	0.018	0.256	0.242
Physical activity ≤3 hours per week	39.9	33.1	35.9	29.6	0.013	0.039	0.202	0.002	0.465	0.354	0.097
Sedentary*	37.5	28.2	32.7	26.7	0.002	0.004	0.123	<0.001	0.203	0.684	0.096
Hypertension	48.2	38.2	31.5	33.5	<0.001	0.002	<0.001	<0.001	0.083	0.260	0.581
Diabetes	14.1	9.0	10.0	6.1	0.001	0.024	0.055	<0.001	0.706	0.153	0.070
Hyperlipidaemia	24.0	14.1	14.5	13.8	<0.001	<0.001	<0.001	<0.001	0.885	0.899	0.786

*Total sitting time ≥7 hours per day+physical activity ≤3 hours per week.

AS, ankylosing spondylitis; BMI, body mass index; CVRF, cardiovascular risk factor; PsA, psoriatic arthritis; RA, rheumatoid arthritis.

Men with gout had higher prevalence of obesity than men with PsA and RA, and more hypertension and hyperlipidaemia than all other IJDs (table 4B). Men with gout more often reported diabetes and a sedentary lifestyle compared with men with PsA and AS. Accordingly, men with gout had higher levels of ≥1, ≥2 and ≥3 trad-CVRFs compared with other IJDs (figure 2B).

The occurrence of obesity, hypertension, diabetes, hyperlipidaemia and a sedentary lifestyle was, however, comparable among men with PsA, RA and AS, who in addition had similar levels of multiple trad-CVRFs (figure 2B). Current smoking was more prevalent in men with RA and less common in men with PsA. Educational level was similar among men across IJDs, except for in RA, where education ≤12 years was over-represented.

## Discussion

In this cross-sectional questionnaire study, comparing gout, PsA, RA and AS we found that women and men with gout had significantly more trad-CVRFs compared with other IJDs, both before and after standardising for age. Women with PsA had, however, higher age-standardised prevalence of obesity and hypertension, compared with women with RA and AS, and higher age-standardised occurrence of diabetes and hyperlipidaemia, compared with women with AS. Conversely, in men, the age-standardised prevalence of trad-CVRFs was similar in PsA, RA and AS. Previous studies have reported on an increased prevalence of CVRFs in IJDs compared with the normal population. However, studies comparing CVRFs across different IJDs are less common, and stratification by sex is sometimes,^24^ but often not,^9 25–27^ presented. This study highlights the strong over-representation of CVRFs in gout. To the best of our knowledge, no study has included gout in the comparison of CVRFs in IJDs. Our results also underline that differences between sexes exist regarding CVRFs among patients with PsA, RA and AS, in particular for women with PsA. This is in line with Swedish register-derived results, which have shown a relatively high risk of CVD among women with PsA.^2^

Obesity has been reported as a risk factor for developing gout^28^ and PsA.^29^ In our study, women and men with gout reported the highest age-standardised prevalence of obesity. Women with PsA were more frequently obese compared with women with AS and RA. Similar to our findings, earlier studies have reported higher frequencies of obesity in PsA compared with RA^25–27^; these studies were, however, not stratified by sex, although the study by Bhole *et al* included sex as a covariate in a multivariable logistic regression, showing an increased risk of obesity in women with PsA compared with men with PsA.^26^

Smoking has been suggested to play a part in the pathogenesis of ACPA-positive RA,^30^ whereas smoking as a risk factor for developing PsA is more unclear.^31^ Incident AS has been associated with current smoking in a large population-based case–control study,^32^ and smoking is associated with increased syndesmophyte formation in men with AS.^33^ In this study, we found that smoking was more common in patients with RA, but again there were differences among women and men. In women, current smoking was comparable in gout, PsA and RA, but significantly less common in AS, whereas in men current smoking was more prevalent in those with RA, who also reported the lowest level of education. This is slightly different from findings from a Norwegian study by Wibetoe *et al* who found no differences in current smoking status between the investigated IJDs, except for in older patients with PsA, where smoking was less frequent.^25^ The study design or true variations between countries may account for this subtle difference in results, and both studies would have benefited by inclusion of a general population comparator.

Having a sedentary lifestyle was more common in women and men with gout, followed by RA, PsA and AS. In women, as well as in men, after standardising for age, a sedentary lifestyle was equally common in PsA, RA and AS. A meta-analysis assessing and quantifying the association between daily sitting time and mortality reported a non-linear relationship where the increase in all-cause mortality was highest for individuals with a total daily sitting time of >7 hours per day, after adjusting for physical activity.^23^ Earlier studies have shown that patients with spondyloarthritis (SpA) and RA may be less active compared with healthy controls.^34 35^ To the best of our knowledge, no previous study has compared living a sedentary lifestyle for gout, PsA, RA and AS simultaneously in the same setting.

Urate may play a pathogenic role in developing hypertension, through contributing to endothelial dysfunction.^36^ Urate is frequently elevated in patients with psoriasis^37^ and possibly contributing to the burden of hypertension in this patient group. We found hypertension to be the most commonly reported trad-CVRF. In women, after standardising for age, hypertension was equally common in gout and PsA, and both were significantly higher compared with RA and AS. In men, patients with gout presented higher prevalence of hypertension compared with all other IJDs. In line with our findings, Wibetoe *et al* reported hypertension as the most prevalent CVRF in all IJDs (present in 49.8% of patients with IJDs).^25^ They also reported that hypertension was more prevalent in PsA compared with RA and axial SpA, whereas another, older study reported similar age-adjusted and sex-adjusted prevalence of hypertension in patients with PsA, RA and AS.^9^

When it comes to diabetes occurrence, after standardising for age, again, both women and men with gout presented the highest prevalence of diabetes. This could be attributed to the high prevalence of obesity in gout, but perhaps partly also because of the role of urate as an enhancer of insulin resistance. Women with PsA and RA had similar prevalence of diabetes, but women with PsA had higher prevalence than women with AS. In men, there were no significant differences in PsA, RA and AS. The diabetes prevalence has previously been reported as similar among PsA, axial SpA and RA by Wibetoe *et al*.^25^ Han *et al* also found similar prevalence of diabetes for PsA, RA and AS.^9^ In contrast to our study, Labitigan *et al* found that patients with PsA more frequently had diabetes compared with patients with RA,^27^ although their PsA cohort had a markedly higher proportion of obese subjects compared with our PsA cohort (45% vs 23%). Also, the study by Castañeda *et al* showed higher prevalence of diabetes and obesity in PsA compared with RA and AS.^24^

In our study, 10.9%–32.1% of patients reported hyperlipidaemia, most frequent in patients with gout and least frequent in those with AS. After standardising for age, we found that both women and men with gout more frequently reported hyperlipidaemia compared with other IJDs. Women with PsA more frequently reported hyperlipidaemia compared with women with AS. No significant differences were seen comparing men with PsA to men with AS.

The prevalence of hyperlipidaemia has previously been reported, ranging from 27.0% to 35.6% for PsA, RA and AS, with the highest occurrence reported in patients with PsA.^24^ Others have found similar prevalence of hyperlipidaemia among IJDs.^9 25^ In contrast to our findings, a study by Labitigan *et al* found that patients with PsA more frequently had hyperlipidaemia compared with patients with RA.^27^ There are many possible explanations for the relatively low prevalence of hyperlipidaemia in our study, such as infrequent lipid measuring in routine healthcare, under-reporting, different definitions used for hyperlipidaemia and different cut-off values used.

In support of the higher prevalence of trad-CVRFs in patients with gout, both women and men with gout more frequently reported use of antihypertensives, antidiabetics and lipid-lowering drugs compared with other IJDs. The only difference seen in education was lower levels in men with RA, suggesting that the over-representation of CVRFs in gout might not be explained by differences in education or socioeconomic status. In this study, we found that multiple trad-CVRFs were more common in women and men with gout compared with other IJDs. Women with PsA had higher prevalence of multiple trad-CVRFs compared with women with RA and AS, whereas among men the prevalence was similar in PsA, RA and AS. The over-representation of multiple trad-CVRFs in women and men with gout and women with PsA is likely partly explained by the higher prevalence of obesity in these groups. Central obesity, insulin resistance and increased levels of circulating fatty acids are suggested mechanisms that underlie the MetS, which comprises a set of cardiovascular risk factors that cluster together—enlarged waist circumference, elevated fasting glucose, hypertension and deranged blood lipids with elevated triglycerides and lowered high-density lipoprotein cholesterol.^38^ Although less recognised, high urate levels have been shown as a predictor for MetS.^39^ Obesity and other comorbidities can reduce the ability to stay physically active, giving rise to a vicious cycle. Obesity may also be involved in the pathogenesis of both gout and PsA and adds to the burden of the diseases. These associations are supported by Mendelian randomisation studies in both gout^40^ and psoriasis.^41^ Gastric by-pass surgery has been shown to reduce the risk of gout.^42^ Obesity and MetS are associated with increased disease activity in PsA.^43^ Co-occurrence of multiple CVRFs has previously been reported as more common in PsA compared with RA and AS, and as many as 73.6% of patients with IJDs have been reported to present at least one CVRF.^25^ A Swedish register study, comparing PsA, AS and undifferentiated SpA, showed an increased risk of acute coronary syndrome and stroke, especially in women with PsA, compared with the normal population.^2^ This further emphasises the need to address trad-CVRFs, especially in patients with gout and in women with PsA.

## Limitations

This study has several limitations that should be mentioned. First, the data are self-reported and may be subject to over-reporting and under-reporting; however, any misreporting is likely to be similar in all IJDs. Second, due to the cross-sectional design of the study, we cannot exclude that the presence of an IJD can lead to increased awareness of and screening for comorbidities, as well as an increased number of healthcare visits, which could lead to increased detection of trad-CVRFs. These effects are, however, likely to be similar for different IJDs, although patients with gout are typically managed in primary care, where screening for CVRFs may be more frequent than in tertiary care. Third, differences in age and sex across diagnoses hampered comparisons, although we already in the design of the study tried to address this by recruiting a relatively higher proportion of men with RA and standardised for age and stratified by sex in the analyses. Fourth, misclassification of diagnoses may be present; however, validation studies of diagnosis of gout,^44^ as well as PsA,^45^ RA^46^ and AS^47^ have shown high validity. Fifth, our study is set in a defined geographical region, Western Sweden, which may limit generalisability to other parts of the country or other countries. However, the area is considered representative of Sweden as a whole with regard to demography and health status.^48^ Also, the non-responders in this study were younger than the responders, and for some diagnoses men were over-represented. In a previous study comparing survey responders with non-responders in patients with RA, the latter were younger and had more severe disease.^49^ Any such effects are present in all IJDs and would therefore not affect the validity of our comparisons. Also, we did not validate and make changes to the questionnaire based on the initial response, which might have been beneficial to identify problematic questions. Furthermore, it would have been beneficial to have a general population control group for external comparison.

## Strengths

A strength of our study is the comparison of four IJDs in an identical setting with regard to geography, time and healthcare system, which has never been done before. In fact, comparisons of trad-CVRF in different IJDs are rare. Second, as knowledge increases about trad-CVRF in IJDs, patterns of these comorbidities may change in IJDs over time, and there is therefore a need for contemporary studies as the present. Third, it is important to gain knowledge regarding the occurrence of trad-CVRF in IJDs, to identify where the need is largest for further interventions against trad-CVRF.

## Conclusions

Trad-CVRFs were over-represented in women and men with gout compared with PsA, AS and RA, even after standardising for age. In women, trad-CVRFs were especially pronounced in PsA. Women with PsA reported higher age-standardised prevalence of obesity, hypertension, diabetes and hyperlipidaemia, compared with women with AS, and higher age-standardised prevalence of obesity, low physical activity and hypertension, compared with women with RA. In men, age-standardised prevalence of trad-CVRFs was more similar in PsA, RA and AS. It is essential to regularly assess and treat CVRFs in patients with IJDs, in particular in patients with gout and in women with PsA, to reduce the burden of disease as well as the risk of CVD.

## Data Availability

Data are available upon reasonable request.
