# Supplementary material for: Cardiovascular risk factors in gout, psoriatic arthritis, rheumatoid arthritis and ankylosing spondylitis: a cross-sectional survey of patients in Western Sweden
Source: RMD Open. 2021 May 23;7(2):e001568. doi: 10.1136/rmdopen-2021-001568 (PMC8154995; doi:10.1136/rmdopen-2021-001568)
Supplement: Supplementary data [file rmdopen-2021-001568supp001.pdf]

Supplementary table 1, Responder- and non-responder data stratified by diagnosis, data compared across diagnoses by chi square tests or t-tests as appropriate

Diagnosis	Responders age, mean (SD)	Non-responders age, mean (SD)	p-value	Male sex, responders (%)	Male sex, non-responders (%)	p-value
PsA	56.6 (13.2)	52.0 (13.8)	<0.001	46.9	51.5	0.118
AS	51.1 (14.8)	44.5 (13.5)	<0.001	56.4	65.6	0.002
RA	66.8 (13.1)	62.7 (15.9)	<0.001	47.8	46.4	0.623
Gout	71.3 (11.9)	69.1 (15.5)	0.001	79.6	67.7	<0.001

PsA=Psoriatic arthritis; AS=Ankylosing spondylitis; RA=Rheumatoid arthritis
SD=Standard deviation
